# Unraveling the Effects of Social Class and Systemic Racism on Advance Care Planning

**DOI:** 10.1093/geroni/igaa057.1528

**Published:** 2020-12-16

**Authors:** Jenny McDonnell

**Affiliations:** Emory University, Roswell, Georgia, United States

## Abstract

While advance care planning (ACP) is recognized as a key facilitator of high-quality, goal-concordant end-of-life care, black Americans are less likely to participate in ACP than non-Hispanic whites (Carr 2011; Detering et al. 2010). There are divided explanations for why these disparities persist. Some scholars attribute racial disparities in end-of-life care to socioeconomic (SES) differences between black and white Americans citing blacks’ and whites’ differentiated access to, control over, and use of material resources (Wilson 1978; Yearby 2011). Others assert that health care preferences do not solely reflect lack of resources or health literacy, but that the larger social context frames care preferences differently across racial and ethnic groups in American society (Alegria et al. 2011; Sewell and Pingel forthcoming). By turning the analytical lens to class-privileged black Americans, I investigate whether racism overflows the margins of class disadvantage. Using data from the Health and Retirement Study, I ran logistic regression and moderation models. I found that class-privileged blacks are less likely to engage in ACP than both high-SES and low-SES whites. The interaction of race and SES was negatively and significantly associated with ACP (OR=0.91; P<0.05), indicating that SES has a stronger effect on the probability of ACP among whites than among blacks. Predicted probabilities show that 51% of low-SES whites are likely to engage in ACP compared to 32% of high-SES blacks. These findings indicate that racialized disparities in ACP exist independent of SES, and that the effects of SES and race are intersectional rather than simply additive.

